# Evaluating the Compliance to 24-Hour Venous Thromboembolism Risk Re-assessment Following Orthopaedic Surgery at a Tertiary Center: A Closed-Loop Clinical Audit

**DOI:** 10.7759/cureus.74882

**Published:** 2024-11-30

**Authors:** Hassan Imtiaz, Tho-Vin Nguyen, Thuy Tuong Vy Thai, Georgios Kouklidis, Majella Horgan, Ramesh Vijayaraghavan

**Affiliations:** 1 Orthopaedics, Royal National Orthopaedic Hospital, London, GBR; 2 Orthopaedics (Joint Reconstruction Unit), Royal National Orthopaedic Hospital, London, GBR; 3 Orthopaedics, University Hospital Dorset, Dorset, GBR; 4 Spinal Surgery, Royal National Orthopaedic Hospital, London, GBR; 5 Anesthesia and Critical Care, Royal National Orthopaedic Hospital, London, GBR

**Keywords:** : clinical audit, nice guidelines, orthopaedic surgery, quality improvement (qi), venous thromboembolism (vte)

## Abstract

Introduction

Venous thromboembolism (VTE) is a preventable cause of patient morbidity and mortality among hospitalised patients. VTE events have a high incidence among orthopaedic patients, who routinely receive chemical thromboprophylaxis in the form of heparin, warfarin, antiplatelet agents or direct oral anticoagulants. These can be associated with adverse events, most commonly bleeding or heparin-induced thrombocytopenia. A VTE risk reassessment following 24 hours of admission or a change in clinical condition like surgery is recommended to avoid such complications. We evaluated the compliance to completion of these reassessments following surgery at a tertiary elective orthopaedic hospital.

Methods

A closed-loop audit was undertaken for all elective orthoapedic specialties. First loop was conducted between 01/07/2022 and 25/05/2023, whilst the second loop was done between 01/01/2024 and 01/02/2024. Insight, an online database was used to collect data on initial VTE assessment forms completed at admission and VTE reassessment forms completed within 24 hours of surgery, as agreed with orthopaedic teams. Audit standards were derived from National Institute for Health and Care Excellence (NICE) guidelines on venous thromboembolism in over 16s. A target compliance was agreed at 100%. Paediatric patients, day case procedures and medical admissions comprised the exclusion criteria.

Results

For the first audit loop, only 6/4780 (0.13%) patients had completed VTE reassessment forms within 24 hours following surgery. Following implementation of a system prompt on Electronic Patient Medication Administration (EPMA) to serve as a reminder for VTE reassessment completion, the second audit loop found 112/156 (74%) patients had completed forms (p<0.001).

Conclusion

VTE risk reassessment following surgery is recommended by NICE guidelines in order to assess and potentially minimize complications like bleeding and heparin-induced thrombocytopenia. A simple intervention such as a system reminder can serve to improve compliance. This can be implemented at a large scale given most hospitals use integrated electronic medication administration systems where initial VTE risk assessments are completed as part of the initial patient admission process.

## Introduction

Venous thromboembolism (VTE) entails two closely related clinical conditions, namely deep vein thrombosis (DVT) and pulmonary embolism (PE) [1]. DVT refers to the development of a thrombus in a vein. If such a thrombus breaks off and dislodges to the pulmonary vasculature, the resultant condition is described as a PE. This can be prevented by timely measures for hospitalised patients, in the form of mechanical or chemical thromboprophylaxis. 

VTE is a leading cause of morbidity and mortality among hospitalised patients. 14,846 deaths attributable to VTE-related events were reported between 2021/2022, within the 90-day period following hospital discharge [2]. The United Kingdom House of Commons Health Committee report estimated that 25,000 patients die from preventable hospital-related VTEs per year [3].

ENDORSE, a large multi-center study, estimated 64.4% of surgical patients were at high risk of developing VTE events [4]. The incidence rates of VTE events in surgical patients are estimated at 0.71% and 0.33% for DVT and PE respectively, with a positive relation between duration of surgery and incidence [5]. In view of this, NHS first mandated data collection on VTE risk assessment in June 2010 on a quarterly basis in order to evaluate compliance and improve any deficiencies [6]. Orthopaedic surgery poses a higher risk of developing VTE. A large prospective study evaluating 45,968 major orthopaedic surgery patients estimated post-operative VTE occurrence at 1.1% [7]. 

Given the adverse outcomes related to VTE events for hospitalised patients, the National Institute for Health and Care Excellence (NICE) guidelines were introduced which recommended that all patients being admitted to hospital should have VTE risk assessment done as soon as possible, and ideally be initiated on appropriate VTE prophylaxis as part of the initial risk assessment [8]. 

As important as this initial chemical thromboprophyalxis prescribing is to prevent VTE, it is equally important to recongnise the potential complications associated which include bleeding and heparin-induced thrombocytopenia. A large retrospective analysis of patients undergoing major orthopaedic surgery reported an incidence of 11.13-12.58% for severe bleeding following administration of fondaparinux or low molecular weight heparin [9]. A meta-analysis of randomized trials for patients undergoing orthopaedic surgery reported a probability of 5% and 19% for major and minor bleeding episodes associated with unfractionated heparin given as thromboprophylaxis [10]. A randomised trial demonstrated that major bleeding complications associated with pharmacological thromboprophylaxis in hip or knee arthroplasty could have an incidence of up to 2.8% [11]. 

Thus, NICE guidelines also recommend that patients should undergo a reassessment of the VTE risk at 24 hours after admission, following surgery or if the clinical condition changes significantly [8]. This ensures that appropriate VTE thromboprophylaxis is prescribed, and it is adjusted or withheld if any adverse outcomes occur, such as bleeding or heparin-induced thrombocytopenia. VTE risk reassessment therefore is an important aspect of patient safety.

Like most hospitals in the UK, our institute ensures that initial VTE risk assessment is completed at admission by ensuring it serves as a limiting step on Electronic Patient Medicine Administration (EPMA), which is used for prescribing patient medications. Without the initial VTE assessment form completed, the system does not allow the user to proceed with prescribing. 

This ensures a high compliance to initial VTE risk assessment completion. EPMA also has a section where VTE risk reassessment forms can be completed. But, there do not seem to be any system prompts or reminders for completing these forms, and therefore, they are not routinely completed by healthcare staff. 

Taking this into account, we aimed to conduct a closed-loop audit to assess the compliance to completion of VTE reassessment forms within 24 hours following surgery by healthcare staff at our institute.

## Materials and methods

A closed-loop audit evaluating the compliance to VTE risk reassessments done following orthopaedic surgery was conducted at a tertiary care orthopaedic hospital. Data was collected retrospectively for both audit loops. The first loop looked at patient data between 01/07/2022 and 25/05/2023, whilst the second loop evaluated patients between 01/01/2024 and 01/02/2024. 

The standard for the clinical audit was derived from NICE guidelines on VTE risk assessment [8]. A target compliance of 100% for VTE risk reassessment form completion was agreed upon among the responsible teams. A cut-off time set for these forms to be completed was agreed upon to be within 24 hours postoperatively.

For the purpose of this study, data was collected retrospectively from Insight, an online database directory of patient records. Insight software is integrated with electronic patient records and EPMA, and pulls through anonymised data on various inpatient documents like VTE forms giving the time and date when these were completed. The parameters evaluated were; initial VTE risk assessment at admission, and subsequent postoperative VTE risk reassessment done within 24 hours of orthopaedic procedure. VTE reassessment forms completed beyond the 24-hour period cut-off were included with unfilled forms in the statistical analysis, as all these numbers indicate non-compliance. 

All orthopaedic subspecialties were included in the study, except orthopaedic trauma (our institute is an elective center). Paediatric population (<12 years of age), day case surgery cases, and those patients not undergoing a surgical procedure met the exclusion criteria. These specific patient populations were excluded as they are either not routinely given VTE (paediatric population) or the inpatient stay is less than 24 hours (day case surgery). 

The findings of the first loop were presented at a local clinical governance meeting, and based upon the findings, it was agreed upon to introduce a system prompt on EPMA, whereby, at 24 hours following surgery, an automatically generated system reminder would come up on screen each time the inpatient medication chart on EPMA was viewed by any healthcare staff. The system prompt appeared as a pop-up screen on EPMA, stating that the patient is within the 24-hour post-surgery period and needs a VTE risk reassessment form completion. Alongside, were tabs that would direct straight to the VTE risk reassessment form or allow the system-generated prompt to be closed if it was not applicable. Once the form was filled, the reminder would not appear again for the same patient. This did not require any training for healthcare staff and relied solely on automated notifications. 

Following this change, the second loop of the audit cycle was conducted to evaluate improvement in this domain. A shorter duration of data evaluation was chosen for the second loop in order to evaluate the initial results following the intervention, to assess its effectiveness, which would guide the need to continue it or aim for a different approach to improve compliance. 

Data was collected and analysed in Excel (Microsoft, Redmond, WA, USA). A Chi-square test was used to test for statistical significance. A P-value of <0.05 was considered statistically significant. 

## Results

First audit loop

Between 01/07/2022 and 25/05/2023, the total number of ‘Initial VTE assessment’ forms completed was 6030. Out of these, 1250 met the exclusion criteria (medical admissions and day surgery cases). Therefore, the total number of cases audited was 4780. 

The total number of ‘VTE reassessment' forms completed during this period was 63. Out of these, only six were ‘24-hour reassessment’ forms. The rest were not filled within a 24-hour period post-procedure, and therefore were rendered non-eligible for consideration. Results are given in Table 1.

**Table 1:** First Audit Loop results VTE: Venous thromboembolism

	Total	Excluded	Total audited
‘Initial VTE assessment’	6030	1250 (medical admissions/day surgery)	4780
‘VTE reassessment’	63	57 (not filled 24-hrs post-op)	6

The results gave an overall compliance of 0.13% for completion of VTE risk reassessment forms within 24 hours following surgery (% Compliance = VTE reassessment / Initial VTE assessment = 6/4780 x 100 = 0.13%).

Intervention to improve practice

A system prompt was initiated on EPMA, which appeared on screen following surgery, every time a healthcare professional accessed the patient medication record, to serve as a reminder that patient requires a VTE risk reassessment form to be completed. 

Second audit loop

Following initiation of a system prompt on EPMA, the second loop for the audit was conducted between 01/01/2024 and 01/02/2024. A total of 156 eligible patient records were evaluated. All of them had initial VTE risk assessments completed at admission. One hundred thirty-one of 156 (83%) patients had a VTE risk reassessment form completed. One hundred twelve of 156 (74%) had the forms completed within 24 hours following surgery.

Therefore, our compliance to 24-hour postoperative VTE risk reassessment went up from 0.13% from the first loop to 74% (p < 0.0001) following the implementation of a system reminder prompt on EPMA. The statistical analysis (contingency table, Chi-squared test) for significance is given in Table 2. 

**Table 2:** Second Audit Loop results and Statistical Analysis * included the 57 forms that were filled after 24 hours **Chi-squared test VTE: Venous thromboembolism

	Completed VTE reassessment forms (within 24 hours post-operatively)	Not completed (n)	Total patients evaluated (n)	p-value**
First Audit Loop	6	4774*	4780	-
Second Audit Loop	112	44	156	<0.0001

## Discussion

This clinical audit highlights the suboptimal compliance to VTE risk reassessment following orthopaedic surgery. With the introduction of a system prompt on EPMA to serve as a reminder, we managed to achieve significant improvements in this regard. 

There is a general trend of low compliance to low VTE risk reassessments. An audit of 400 orthopaedic patients reported 0% of these assessments completed [12]. A similar assessment for general surgical patients had a compliance of 15% [13]. A more broad study covering inpatients across different surgical specialties assessed the baseline compliance as 32.9% for VTE risk reassessments [14].

Implementation of reminders on electronic patient records has been documented to show improvements in compliance with VTE risk assessments [14]. This being said, this report is among the first to put forth the concept of a VTE risk reassessment reminder by integration with an electronic patient medication administration system, and the significant positive impact it can achieve.

Given that a rising trend towards electronic patient records and medicine administration is underway, we anticipate that most hospitals in the future would utilize this approach. Currently, most hospitals in the UK use EPMA which incorporates the initial VTE risk assessment forms as mandatory steps prior to allowing prescription of medications. This ensures the target of 95% compliance for these forms within the first 14 hours of admission, as set by NHS Standard Contract is achieved by UK hospitals [15].

In view of the fact that most UK hospitals utilise an electronic medication prescription system similar to EPMA, this report can serve as a tool for other institutes to employ and achieve better compliance rates to VTE risk reassessment. In institutes where such systems are not utilised, the concept of a reminder to healthcare staff to fill out these forms can be conducted by means of either highlighting patients within the 24-hour post-operative period on healthcare staff handover sheets, or by placing posters in wards to grab the attention of relevant team members. 

The study limitations include a smaller sample size in the second audit loop due to the fact that this loop was conducted shortly after implementing the system prompt on EPMA. This gave a shorter time frame and therefore a smaller patient population for study. Conducting the second loop early after an intervention is implemented also leads to a potential limitation, that is it cannot indicate whether the changes seen will be sustained, as change of healthcare staff usually occurs over a short period of time, and therefore, it needs a re-evaluation further down in time. Another limitation was the lack of subjective input from healthcare professionals pertaining to challenges with complying to VTE risk reassessment completion. This could provide insights into the suboptimal performance during the first audit loop.

To ensure whether the improvements achieved following implementation of a system prompt are sustained, a further audit loop is recommended. 

## Conclusions

VTE risk reassessment following a change in clinical situations such as undergoing surgery is a key safety measure that needs much better compliance in order to avoid adverse events following use of pharmacological thromboprophylaxis. This requires attention from hospital VTE committees and patient safety departments to employ adequate measures to prevent avoidable patient harm. This report presents a novel approach to address this issue by the implementation of system-integrated VTE risk reassessment reminders at clinically relevant and frequently encountered checkpoints, such as electronic medication administration systems. It also highlights that steps should be taken to implement a strategy similar to the one existing for initial VTE risk assessments at admission across the healthcare sector to ensure greater compliance with subsequent VTE risk reassessments. 
